# A Semantic Framework to Detect Problems in Activities of Daily Living Monitored through Smart Home Sensors

**DOI:** 10.3390/s24041107

**Published:** 2024-02-08

**Authors:** Giorgos Giannios, Lampros Mpaltadoros, Vasilis Alepopoulos, Margarita Grammatikopoulou, Thanos G. Stavropoulos, Spiros Nikolopoulos, Ioulietta Lazarou, Magda Tsolaki, Ioannis Kompatsiaris

**Affiliations:** 1 Information Technologies Institute, Centre for Research & Technology Hellas, 6th Km Charilaou-Thermi, 57001 Thessaloniki, Greece; ggiannios@iti.gr (G.G.); lamprosmpalt@iti.gr (L.M.); valep@iti.gr (V.A.); marggram@iti.gr (M.G.); athstavr@iti.gr (T.G.S.); iouliettalaz@iti.gr (I.L.); ikom@iti.gr (I.K.); 2 Department of Neurology I, Medical School, Aristotle University of Thessaloniki, 54124 Thessaloniki, Greece; tsolakim1@gmail.com; 3 Greek Association of Alzheimer’s Disease and Related Disorders (GAADRD), 54643 Thessaloniki, Greece; 4 Laboratory of Neurodegenerative Diseases, Center for Interdisciplinary Research and Innovation (CIRI-AUTh), Balkan Center, Buildings A & B, 57001 Thessaloniki, Greece

**Keywords:** smart home, sensors, ADLs, Semantic Web, ontology, knowledge graph, SPARQL rules

## Abstract

Activities of daily living (ADLs) are fundamental routine tasks that the majority of physically and mentally healthy people can independently execute. In this paper, we present a semantic framework for detecting problems in ADLs execution, monitored through smart home sensors. In the context of this work, we conducted a pilot study, gathering raw data from various sensors and devices installed in a smart home environment. The proposed framework combines multiple Semantic Web technologies (i.e., ontology, RDF, triplestore) to handle and transform these raw data into meaningful representations, forming a knowledge graph. Subsequently, SPARQL queries are used to define and construct explicit rules to detect problematic behaviors in ADL execution, a procedure that leads to generating new implicit knowledge. Finally, all available results are visualized in a clinician dashboard. The proposed framework can monitor the deterioration of ADLs performance for people across the dementia spectrum by offering a comprehensive way for clinicians to describe problematic behaviors in the everyday life of an individual.

## 1. Introduction

Activities of daily living (ADLs) are essential and routine tasks that most healthy individuals can perform without assistance. ADLs are divided into two major categories: basic ADLs (BADLs) and instrumental ADLs (IADLs) [1]. BADLs, also known as physical ADLs, are a category of daily activities required of each individual to manage their basic physical needs. These activities include self-feeding, dressing, toileting, ambulating and any other physical action that is related to personal hygiene. On the other hand, IADLs are more complex activities that people perform in order to be functional and independent within society. Managing finances and medications, food preparation, housekeeping and laundry are only some elementary examples in this category. Based on the literature, cognitive and functional decline first becomes tractable when performing IADLs due to their complexity [2]. Most people who show early signs of cognitive decline tend to develop an abnormal behavior that makes them deviate from the correct execution of ADLs. This weakness is exacerbated gradually, and it is a common phenomenon that patients who exhibit such cognitive decline do not perceive the occurred deviation [1]. The inability to accomplish essential activities of daily living may be an indicator of an upcoming health problem [1,3]. Investigating the accuracy of daily performed activities can contribute to tracking the health deterioration of people with chronic diseases such as dementia or multiple sclerosis. Engaging in monitoring ADLs is motivated by the need to have an objective way to assess whether there is a diminishing ability to perform certain tasks, compared to or as a ground truth measure of an individual’s self-reporting. That process can offer meaningful insights to clinicians when assessing ADLs to enhance patient care and management.

The ADL assessment process could be classified into several stages in which clinicians look for different types of problems. Any observations or conditions during the execution of ADLs that deviate from a reasonable and normal situation can be considered problems. The repeated occurrence of abnormal behaviors, like the need for excessive time to complete an ADL and difficulty in comprehending instructions on the execution of a specific ADL when those are provided, is only one of the many factors to be evaluated by clinicians. The complexity of monitoring and assessing ADLs imposes the need to facilitate this procedure through the use of sensor technology in a semantic framework.

There is a variety of affordable and accurate devices in the market that can be used to monitor patients by collecting their daily life data [4]. The collected data could be processed and analyzed in order to reflect activities of daily living and allow for valuable insights into patients’ behavioral patterns. For example, smartphone apps and wearable sensor devices, such as smart watches, have been widely used to monitor heart rate, steps and sleep stages [4,5]. Another such category of devices is sensors that capture presence and motion in a room. Consequently, there is a significant need for the establishment of a framework that would integrate, analyze and visualize all available data in an efficient way. Such a framework would support the evaluation of the data received by expert clinicians allowing them to draw useful conclusions. The introduction of the Semantic Web (Web 3.0) [6] and its constantly developing technologies, such as ontologies, RDF (resource description framework) [7], SPARQL [8], reasoning engines and triplestores, provide us with all the essential tools to construct a framework that could implement all the processes mentioned above.

In this paper, we propose a semantic framework for detecting problems in ADLs monitored through smart home sensors. This framework consists of different components that are responsible for monitoring and modeling ADLs, executing specific rules to detect problematic behaviors in ADLs performance and visualizing the results. In this way, we can detect problems related to cognitive decline and observe the scale of a patient’s health deterioration within a given period of time. Initially, we gather all raw data provided from sensors and devices installed in a smart home environment. Next, a specially adapted ontology is developed to model and represent all the related information of this environment. This includes information and data acquisition from patients, sensors, devices and rooms of the smart home. Then, all collected data are converted to RDF triples and stored in a semantic database constituting a knowledge graph. Furthermore, by using SPARQL queries, we define and construct explicit rules to detect problems, a procedure that leads to generating new implicit knowledge. Last but not least, all available results are visualized in a clinician web dashboard.

The rest of the paper is structured as follows: Section 2 reviews the related work regarding the use of Semantic Web technologies for e-health solutions. Section 3 describes the architecture of the proposed framework, illustrating all the background processes. Section 4 presents a case scenario providing visualized results. Finally, Section 5 draws our conclusions and describes our intentions for future work.

## 2. Related Work

The proposed framework uses techniques from multiple research fields, including Semantic Web Technologies, ADLs recognition and monitoring through smart home sensors. In this section, we examine previous related studies that incorporate these technologies and methods to provide e-health solutions.

Passive remote monitoring systems have the potential nowadays to collect data for longitudinal research on daily activity, cognitive function, or treatment response. Smart home technology, as proposed in a recent literature review, monitors both cognitive functions and ADLs in a simulated home environment [9], while the installed sensors can detect potential risks and enhance remote clinical assessment [10]. Therefore, adapting assistive technologies like smart homes to support independent living for people with dementia or early stages such as mild cognitive impairment (MCI), offering applications for memory aid, executive functions, social interactions, and more, constitutes a challenging recent approach [11]. Several research studies have so far been used to capture daily vital signs and automatically detect ADLs, enhance autonomy, recognize emergencies, and track the disease progression of individuals with cognitive impairment [12,13,14] through smart home systems.

On the other hand, specific systems have been designed to provide support for elders dealing with time-related issues [15,16], difficulties in spatial orientation [17,18,19], and psychological support due to social isolation and emotional instability [20]. Additionally, various prompting devices have been developed to assist individuals with memory disturbances in their daily lives [21]. In a broader context, several technical solutions have emerged to address the needs of people with cognitive impairment, including web-based information systems, video-calling services, and electronic activity support systems [22,23,24]. Nevertheless, taking a more comprehensive approach, smart homes have demonstrated their utility using combined sensors and several technologies in a home-simulated environment in managing cognitive impairment associated with Alzheimer’s disease (AD) [25]. Thus, in AD-related research, smart homes that incorporate a range of sensors (such as accelerometers, microphone arrays, pressure-sensitive mats, gas sensors, etc.) have been proven effective in monitoring users’ ability to handle ADLs, improving quality of life [26,27] and preventing significant accidents.

Furthermore, a variety of technological methods and solutions have been introduced for the assessment and detection of ADLs and behaviors. These approaches aim to identify patterns and problems and facilitate early detection, incorporating a graded hierarchy of technology-based prompts, statistical and knowledge-based hybrid methods, as well as machine learning paradigms applied to sensor data [28,29,30]. In this work, the proposed system combines different Semantic Web technologies (i.e., ontology, triplestore, SPARQL) to handle and transform smart home sensor data into meaningful representations. Compared to similar technological solutions, the proposed system provides a solution for clinicians, offering (a) knowledge reusability based on the implemented ontology, (b) transparency and interpretability compared to AI models, (c) the option to construct rules of diverse complexity.

In the context of Semantic Web technologies and frameworks, similar solutions have been previously introduced that use ontology-based knowledge graphs for rule-based detection in chronic diseases like multiple sclerosis [31], cancer [32], and deafblindness [33]. In [34], a similar rule-based framework for the detection of health-related problems of people in the spectrum of dementia is presented, using a complete semantic system, including IoT data collection, knowledge Ingestion, as well as a set of SPARQL inferencing notation (SPIN) rules to infer problems from collected data. IoT data collection is the process of collecting data from sensors connected to the Internet Of Things (IoT), which is the interconnected network of physical devices capable of exchanging large amounts of data [35]. Our work uses a similar approach, using IoT data and a rule-based framework to detect ADL problems in a smart home setting. It provides a user-friendly solution and is highly scalable, facilitating easy expansion through the integration of additional IoT sensors. This scalability not only enhances its adaptability but also enables the support of diverse rule creation for various scenarios, thereby amplifying its overall functionality and applicability. Therefore, the proposed framework provides a powerful tool to clinicians for detecting problems in ADL execution and can offer valuable insights into the progression of cognitive decline.

## 3. Semantic Framework

**Figure 1:**
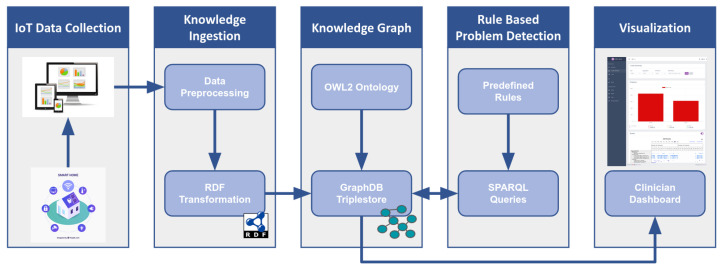
Architecture of the proposed framework.

This section presents the proposed semantic framework for monitoring ADLs in a smart home environment with the aim of detecting problems related to their performance. An overview of the architecture of this framework is shown in Figure 1. The first stage of the pipeline is the IoT data collection from sensors in a smart home setting to monitor ADLs. The next stage includes the pre-processing of the raw data and their conversion to RDF triples. The combination of RDF data and the implemented ontology that defines their structure constitute the knowledge graph. Afterward, predefined rules in SPARQL format are applied to the knowledge graph, aiming to detect problems in ADLs execution. Finally, the visualization of the results in a clinician dashboard concludes this procedure.

### 3.1. Monitoring of ADLs in a Smart Home Environment

For the purposes of the present work, the CERTH-ITI smart home premises were used [36]. The smart home is a prototype and novel technologies demonstration infrastructure resembling a real domestic building where occupants can experience actual everyday life scenarios. Alongside their accommodation, they can explore various innovative smart IoT-based technologies provided with energy, health, big data, robotics and artificial intelligence (AI) services. In the context of a pilot study, 40 participants with varying cognitive status across the dementia spectrum were invited for a daily visit or overnight stay in the smart home. In detail, 25 participants visited the smart home for a day, while 15 participants additionally stayed overnight and left the smart home the next morning. Prior to their enrollment, a neuropsychiatrist with expertise in dementia diagnosed the participants (13 Healthy Controls—HC, 14 participants with Subjective Cognitive Decline—SCD and 13 participants with Mild Cognitive Impairment—MCI). Diagnoses were set, taking into account the participant’s medical history, structural magnetic imaging (MRI) and a detailed neuropsychological evaluation. The MCI group fulfilled the Petersen criteria [37], while the SCD group met IWG-2 Guidelines [38], as well as the SCD-I Working Group instructions [39]. They were instructed to execute a protocol including three ADL activities (namely, Task 1—Hot Meal Preparation, Task 2—Hot Beverage Preparation and Task 3—Cold Meal Preparation). The protocol was accompanied by a detailed, step-by-step description for each Task. For example, in Task 3, participants were instructed to grab a plate, bread, cheese and turkey from appropriately labeled cabinets and the fridge and prepare their sandwich. Afterward, participants were instructed to turn on the appliance, place the sandwich on the toaster and remove it once ready. In order to monitor the participants’ ADLs, we installed a set of smart home devices, including multiple types of sensors that perform functions such as monitoring, controlling or alerting. For our research purposes, we equipped the smart home environment with wall plugs and motion, door, drawer and cabinet sensors. Wall plugs are consumption monitoring devices in which all other electrical appliances are plugged. For example, wall plugs were placed to monitor the kettle, toaster and hot plate consumption—appliances crucial for the performance of the ADLs. Motion sensors are responsible for capturing presence or movement in a room, and door sensors detect the opening and closing of doors, drawers and cabinets. It is noted that the motion sensors were positioned according to the sensor’s manual in the room’s corners and perpendicular to the doors. Through testing, it was ensured that the detection range of the sensors was adequate, covering the study’s need to monitor the presence in each room. For safety reasons, a panic button sensor was also installed so as to provide a quick way to trigger an alarm in case of emergency. It is important to mention that there are many solutions in the market for smart home devices that can be used in similar studies. In our study, we selected the “Fibaro” solution [40], which provides its Home Center software (lite version [41]), allowing users to control and collect data from all devices through a single interface. All the above devices are listed in Table 1.

Fibaro’s raw data consists of two time series—one for “Signal” and one for “Consumption”. Signal is generated by the motion, door and panic sensors, while Consumption is generated by wall plugs. Both the Signal and Consumption time series represent changes in the values that occurred at a specific point in time. Signal, in contrast to Consumption, takes into account the previously reported values in addition to the current values.

A Signal data object for a device (motion, door, panic) is a pair of Boolean values that represent its change in state. If a data object has a new value of 1 and an old value of 0, it generates a new Signal and the timestamp of the data object acts as the starting point, while the next data object with opposite values represents the end of this Signal.

Consumption data, on the other hand, are floating-point values of wattage consumption. Most household appliances consume electricity even when they are idle. We have set a minimum threshold of 5 watts to mark the beginning and the end of a consumption event. This is an empirical threshold we set after careful study and analysis of the generated data. If the wattage of the data object exceeds the threshold, we assume that the appliance is switched on, and if the wattage is below the threshold, the appliance is considered “not in use” (i.e., idle).

All sensor data are transferred to our databases through APIs in order to be gathered and processed. In this way, the data are grouped together and can be further processed by conducting validity checks in order to identify and normalize false negative signals (i.e., Cabinet didn’t fully close due to brakes but the sensor failed to recognize it). This procedure of validity checking ensures the reliability of the collected data, which is necessary for their analysis.

### 3.2. Sensor Data Knowledge Ingestion

**Figure 2:**
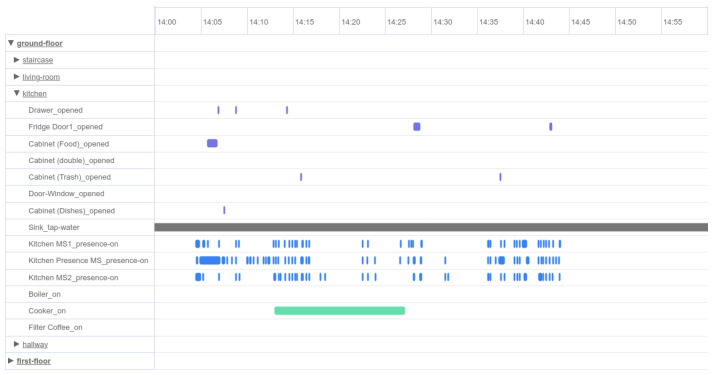
Data visualization dashboard: representation of events related to the kitchen room that have been monitored by sensors.

The raw data described in the previous section are in a complicated form, making them incomprehensible and confusing. Therefore, further analysis is necessary to group them in clusters depending on their timestamps and their source devices. Each cluster consists of raw data that indicate a single event inside the smart home environment. For instance, if a motion sensor in the kitchen detects presence in a specific timestamp while a door sensor detects the opening of a kitchen drawer at the same or subsequent time, then it is reasonable to conjecture that someone was in the kitchen and opened the drawer at that time. By manually inspecting and clustering all raw data into specific meaningful events, we can proceed to the next step of data processing, which is the recognition of an entire activity. Figure 2 is a chart that presents all events derived from raw data in a specific period of time for a specific patient. The names of the events are shown on the y-axis, while the x-axis presents the duration of each one. More specifically, Figure 2 shows a sporadic use of kitchen drawers and cabinets, an almost continuous human presence in the kitchen and the simultaneous operation of the electric cooker for several minutes.

**Figure 3:**
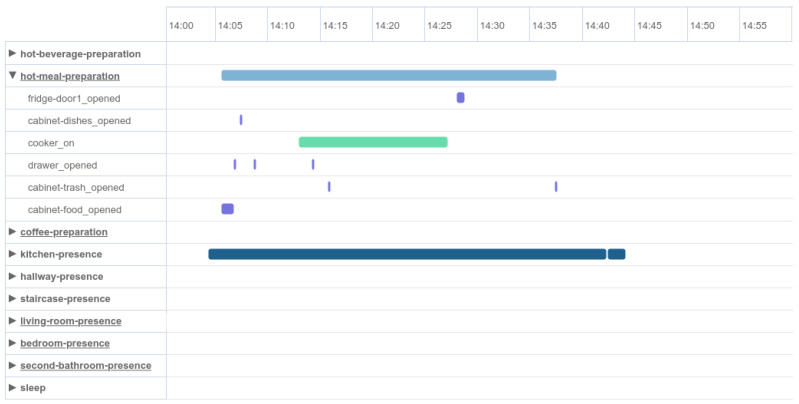
Data visualization dashboard: representation of a hot meal preparation activity consisting of its events.

Following the same procedure, we can create some larger clusters that consist of correlated events corresponding to the room of the smart home and the time series that occurred. For instance, grouping events that occurred in the kitchen, such as motion detection in space, opening and closing of drawers and operation of the electric cooker in a specific period of time, composes a sequence of events for a hot meal preparation activity. This activity is depicted in Figure 3 with the horizontal light blue line. Below that line, some of the previously mentioned events that reveal the cooking activity are included. Similarly, we can identify all different ADLs that patients were instructed to perform based on the protocol during their accommodation in the smart home.

In order to test the effectiveness and reliability of the sensors and the developed platform, the activities collected were compared to ground truth information. For this, prior to the study, researchers performed tests to explore the sensors and the platform’s responsiveness. Furthermore, in order to provide a “quantitative” evaluation, the tasks performed by the participants during the study were used. The ground truth comprised free text notes collected by the researchers and included observations made at the end of the participants’ visit, relying on their feedback along with objective observations made by the researchers. As for each participant, the Smart Home setting was carefully prepared with specific products and portions made available for the protocol execution so their consumption could be monitored (along with, e.g., leftover meals). Discrepancies were noted due to various factors, including power and internet outages, leading to the omission of “Meal Preparation” and “Beverage Preparation” activities. Additionally, there were instances of sensor connectivity issues, resulting in the absence of two “Cold Meal Preparation” activities in the smart home. Specifically, for the “Hot Meal Preparation” activity, the platform recorded 30 activities, while the ground truth indicated 31 instances. Similarly, in the “Hot Beverage Preparation” activity, the platform recorded 31 activities compared to the ground truth, which documented 32 occurrences. In the case of “Cold Meal Preparation” activities, the platform recorded 10 instances, while the ground truth reported 12. These variations highlight the challenges and potential sources of discrepancies in the data caused by external factors impacting sensor functionality.

Having completed all the necessary pre-processing of the raw data, it is imperative to translate our unstructured clustered data into meaningful interlinked graph representations. In this way, our data are linked through meaningful relationships that are humanly understandable, establishing a knowledge base capable of inferring complicated implicit relations. This procedure is called knowledge ingestion (KI) and is achieved through the conversion of our data to RDF statements. An RDF statement is a triplet that links a subject with an object via a predicate. The subject and the object elements represent the two entities that are linked together, and the predicate element strictly defines their relationship. Multiple RDF statements create a larger linked graph that depicts all possible entities and relations that exist in each case we try to model. However, before converting our data to RDF triplets, we have to define a precise schema that all of them should follow.

### 3.3. Ontology-Based Knowledge Representation

In the semantic framework introduced in this paper, it was essential to construct an ontology representing all entities involved in our research. Indicatively, the events and activities that were generated after the pre-processing of the raw data, the smart home sensors, and the patients who participated in the study constitute our primary entities. Therefore, an ontology named “ADL Recognition Ontology” was designed and developed in OWL 2 language with the usage of the open-source software Protégé 5.5.0.

OWL (Web Ontology language) is an ontology language for the Semantic Web with well-defined meaning that can model and represent complex knowledge [42]. In addition, OWL offers more modeling primitives than the RDF Schema and has quite clean formal semantics, which assigns an unambiguous meaning to its logical statements. Formal semantics precisely describe the meaning of a language, which means that semantics can not be interpreted in more than one way. Furthermore, formal semantics are a prerequisite for automatic reasoning support, which is of primary importance since it allows us to check the correctness of our ontology with respect to its consistency and the existence of unintended relations between classes. Moreover, a reasoner is able to infer logical consequences by combining explicit knowledge with the formal semantics of the ontology’s language. In other words, a reasoner generates new relations between the entities that are classified according to the ontology. This mechanism helps us avoid explicitly declaring a vast amount of necessary statements, which we would inevitably have to do if we had a classic relational database schema instead of an ontology.

**Figure 4:**
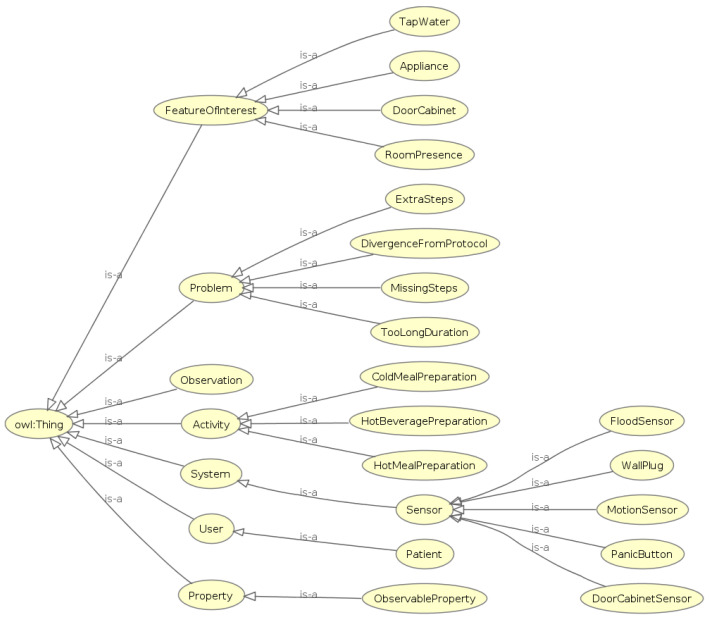
Owl classes of ADL recognition ontology.

Figure 4 shows the OWL classes constituting the “ADL Recognition Ontology”. This ontology is an adapted and extended version of the semantic sensor network (SSN) ontology and the sensor, observation, sample, and actuator (SOSA) ontology [43]. The combination of these two well-established ontologies is intended to provide a comprehensive framework for describing sensors and their corresponding observations. This combined ontology has been used as a fundamental core in other applications [44]. The utilization of these well-established representation models ensures the interoperability of our framework. One of our primary goals was to develop an extensible ontology in order to ensure the flexibility of supporting more scenarios in the future by adding further subclasses to each class category. For instance, the “Sensor” class can represent any other sensor that may be used in a smart home environment. Likewise, this ontology could be enriched with additional “Activity” subclasses depending on the cases being studied. Furthermore, the structure of the “Problem” class gives us the opportunity to add more problem types that may have occurred during the execution of an activity. In our ontology, the “Problem” class has four subclasses, each modeling a specific problem that may be observed by assessing an Activity execution. As mentioned in previous sections, these problem types are related to deviations from the protocol that smart home participants were instructed to follow. After consulting clinicians, we ended up modeling four problem types with the corresponding “Problem” subclasses. These problem types are divergence from the protocol of an ADL, observation of extra or missing steps in each ADL protocol and excessively long duration of an Activity until it is completed.

**Figure 5:**
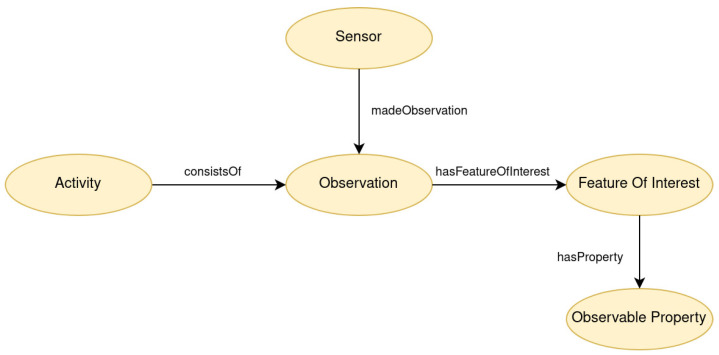
Relations between fundamental SSN/SOSA classes and the Activity class.

Generally, the “Observation” class represents an event that was monitored by a “Sensor” instance. Consequently, the “Activity” class represents an activity that was synthesized by one or more “Observation” instances. Each “Observation” is linked to a specific “FeatureOfInterest” that describes what is being observed or measured by a sensor. In the “ADL Recognition Ontology”, this class is extended with four subclasses that specify the type of sensor that generated each observation. The “ObservableProperty” class is associated with “Observation” class and represents the attributes captured by a sensor. For example, in the case of a sensor capturing a presence in a room, the timestamp and the switch from 0 to 1 value are the attributes of this observation. Figure 5 represents the relations between the “Observation” and the “Activity” class in the ADL Recognition Ontology. The classes “Sensor”, “Observation”, “FeatureOfInterest” and “ObservableProperty”, along with their associated relationships are important components of the SSN/SOSA ontologies. These classes have been expanded with numerous subclasses and linked to new classes to fulfill the representation requirements of our study.

The developed OWL ontology plays a vital role in structuring the data schema within the triplestore, providing a well-organized representation. Its structure allows only fundamental reasoning tasks, focusing on checking ontology consistency and applying straightforward inference rules. This involves making deductions based on properties and ontology hierarchy. Despite the simplicity of these tasks, the ontology significantly enhances schema adaptability and extensibility. Thus, it can be considered as a semantic layer, guiding the interpretation and organization of sensor data. Moreover, OWL allows for more expressive relations between entities compared to conventional database schemas. Leveraging OWL constructs, such as classes, properties, and axioms, facilitates a detailed representation of complex relationships in our study’s domain. This is essential for capturing intricate connections among multiple sensors, various observation types, and participants, resulting in a more thorough understanding of the data. Furthermore, a concept-oriented representation of the domain knowledge with properties and attributes is particularly beneficial for domain experts. This approach provides a more intuitive and meaningful structure for experts to reflect on and define problems within the context of our study, in contrast to a relational-based schema, which might lack expressiveness in capturing relationships.

Both “ADL Recognition Ontology” and converted linked data compose our knowledge graph, which is hosted in an instance of GraphDB, a semantic database offered by Ontotext [45]. GraphDB is a highly efficient and scalable RDF database that can handle massive loads of triples, queries and OWL inferencing in real-time. In addition, it supports all RDF syntaxes for data insertion and retrieval, and it also offers the capability to submit all types of SPARQL queries. One major benefit that led us to choose GraphDB as our triplestore is that it can perform semantic inferencing at scale, allowing users to derive new semantic statements from existing facts due to the existence of an integrated reasoner.

**Figure 6:**
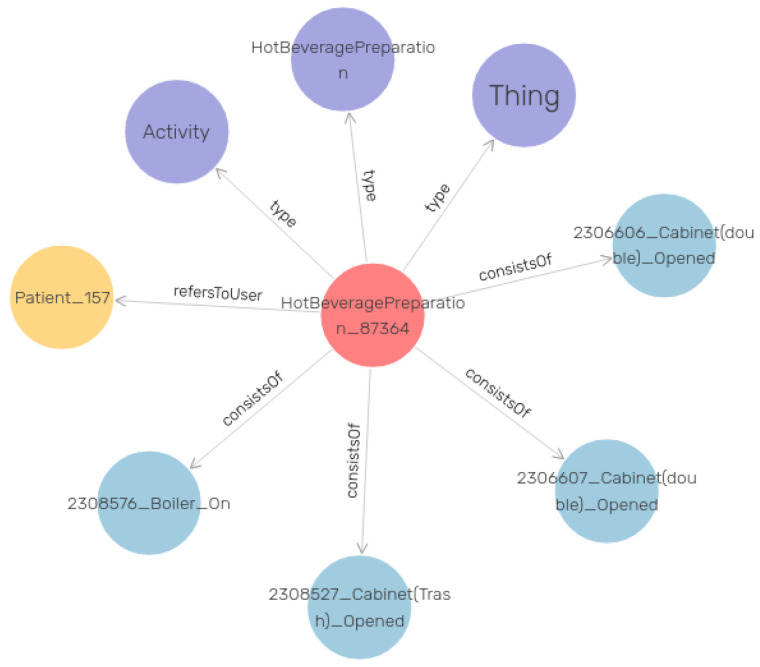
Knowledge graph: visual example of an Activity instance and its relations.

Figure 6 visualizes an example of the RDF data in our knowledge graph. In this example, the central red node is an instance of the “Activity” class as it is described in the purple node. The four edges with the label “consists of” end up in the four different instances of the “Observation” class that constitute the main Activity. In order to better comprehend this graph instance, we can consider the red node as a “Hot Beverage Preparation” activity. The “Observation” objects represent the events that occurred during this activity execution. In this case, these events are the opening of specific kitchen drawers and cabinets, as well as turning on the coffee machine. Last but not least, the yellow node represents the patient that performed this activity. The graph pattern presented in Figure 6 is just one instance of the thousands that are linked together to construct the entire knowledge graph.

### 3.4. Problem Detection Based on Daily Activities

The primary aim of the proposed framework is to be able to detect problems related to the execution of ADLs. To achieve this, we had to define a set of rules as they are presented in Table 2. These rules define upper or lower limits to filter out activities that deviate from a normal execution. The “correct order” and “correct count” variables that appear in the table refer to the predefined protocol instructions that all patients had to follow. The structure of the rules and the numerical values of the thresholds were decided after consultations with clinicians. By setting these thresholds, our intention is not to categorize patients into health groups but to individually monitor their performance and compare it with their former results. In the Semantic Web, these rules can be formulated with SPARQL queries. We used the interface of GraphDB Workbench, an admin web application offered by GraphDB, to compose and execute a set of SPARQL CONSTRUCT graph patterns to derive problematic situations expressed in RDF triples and enrich the knowledge graph.

The following code blocks show an example of the form and structure of SPARQL rules that are responsible for deriving problematic situations in “Hot meal preparation” activities. The first one is a rule for the “Too Long Duration” problem (Listing 1). Applying this query constructs four new RDF triplets that are related to the problem, the patient to whom it is referred, the date that it occurred and the total duration of the problematic activity. In this example, the condition that must be satisfied for the activity is a total duration of more than 2100 s (35 min).

**Listing 1.** SPARQL rule for ‘TooLongDuration’ problem.CONSTRUCT{ ?problem a :TooLongDuration; :isProblemOf ?patient; :problem_date ?start; :problem_rate ?duration. }WHERE { ?activity a :Activity; :activity_pk ?activity_pk; :activity_start ?start; :activity_end ?end; :activity_name ’Hot Meal Preparation’; ;:refersToUser ?patient. BIND((?end - ?start) as ?duration) FILTER( ?duration > “2100”^^xsd:duration ) }

The second rule is for the “Extra Steps” problem (Listing 2). Similarly, it constructs four new triplets describing the problem if the total count of steps needed to complete the activity was higher than 20.

**Listing 2.** SPARQL rule for the ’ExtraSteps’ problem.CONSTRUCT{ ?problem a :ExtraSteps; :isProblemOf ?patient; :problem_date ?start; :problem_rate ?steps. }WHERE { ?activity a  :Activity; :activity_name ’Hot Meal Preparation’; :activity_pk ?activity_pk; :consistsOf ?event; :refersToUser ?patient. BIND(count(distinct ?event) as ?steps) FILTER( ?steps > 20 ) }GROUP BY ?activity_pk ?patient

The third rule implements the “Missing Steps” problem, following the same logic as the previous rules (Listing 3). The condition that must be satisfied in this rule is that the total count of steps needed to complete the activity must be lower than 10.

**Listing 3.** SPARQL rule for the ’MissingSteps’ problem.CONSTRUCT{ ?problem a :MissingSteps; :isProblemOf ?patient; :problem_date ?start; :problem_rate ?steps. }WHERE { ?activity a  :Activity; :activity_name ’Hot Meal Preparation’; :activity_pk ?activity_pk; :consistsOf ?event; :refersToUser ?patient. BIND(count(distinct ?event) as ?steps) FILTER( ?steps < 10 ) }GROUP BY ?activity_pk ?patient

The last rule implements the “Divergence from protocol” problem (Listing 4). This rule helps us comprehend the value of utilizing a powerful language such as SPARQL when implementing a more complex query instead of performing a simple threshold comparison. In this query, we formulate a set of rules that must be strictly satisfied, which refers to the chronological sequence in which certain steps were implemented. More specifically, this rule intends to detect the occurrence of any divergence from the protocol that instructs participants to first open the food cabinet, then the cooker, and lastly, the fridge door. Therefore, this query retrieves all activities that consist of these steps and then checks their time sequences by assigning to them a “correct” or “wrong” status. Finally, it filters out all activities having only correct sequences of these three steps and returns the activities that had at least one divergence from the protocol.

**Listing 4.** SPARQL rule for the ’DivergenceFromProtocol’ problem.CONSTRUCT{ ?problem a  :DivergenceFromProtocol :isProblemOf ?patient; :problem_date ?start; :problematicActivity ?activity. WHERE{ ?activity :consistsOf ?event1; :activity_start ?start; :activity_name “Hot Meal Preparation”; :refersToUser ?patient. ?event1 :observation_start_time ?time1; :refersToDevice ?device1. ?device1 :device_name “Cabinet (Food)”.   ?activity :consistsOf ?event2. ?event2 :observation_start_time ?time2; :refersToDevice ?device2. ?device2 :device_name “Cooker”.   ?activity :consistsOf ?event3. ?event3 :observation_start_time ?time3; :refersToDevice ?device3. ?device3 :device_name “Fridge Door1”.   BIND(IF(?time1<?time2 && ?time2<?time3, “Correct”, “Wrong”) AS ?status) FILTER EXISTS{FILTER(?status!=“Correct”)} }GROUP BY ?activity HAVING (COUNT(?event1) >= 1 && COUNT(?event2) >= 1 && COUNT(?event3) >=1)

### 3.5. Data Visualization Dashboard

**Figure 7:**
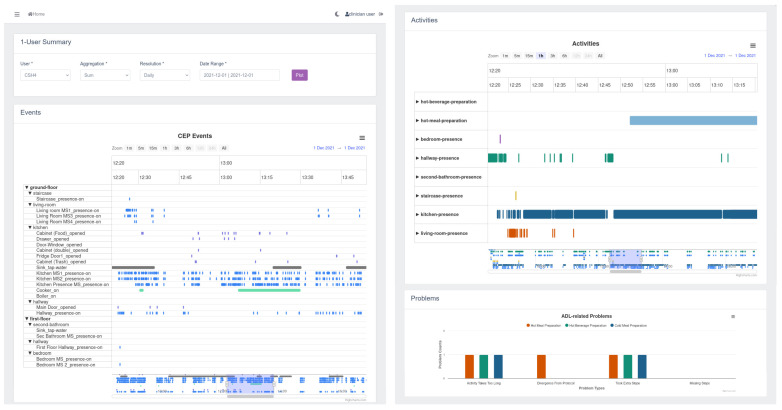
Overview of the data visualization dashboard.

The data visualization dashboard, presented in Figure 7, is a significant element of our framework, providing clinicians with an intuitive and comprehensive overview of the monitored activities and detected problems. This dashboard consists of three main sections: the data filter section, empowering users to apply filters such as participant selection, date range, and resolution; the events and activities section, presenting a detailed log of activities monitored by smart home sensors for each participant; and the problem plot, visually representing the detected problems over time. These components offer clinicians a valuable tool to efficiently analyze and interpret the data collected by our semantic framework.

## 4. Evaluation

The smart home study lasted for a few weeks, so each participant could take part in our research only once. Therefore, we could not monitor participants’ ADLs performance in the long term in order to have enough results to compare them individually. However, for the evaluation of the proposed semantic framework, we present a case scenario in which we examine the procedure of monitoring ADLs, analyzing the data, and detecting problems. Specifically, we focus on the “Hot Meal Preparation” activity, chosen due to its extensive dataset resulting from widespread participant engagement, making it a robust candidate for reliable evaluation.

We gathered and analyzed data from all participants in each health group, including individuals with MCI, those in the HC group, and participants classified in the SCD category, all following the same protocol. After the data collection and analysis, we compared the results of the average duration and steps for the execution of the “Hot Meal Preparation” activity across all participants, as shown in Table 3.

According to the protocol, the maximum normal duration for this activity was set to 2100 s (35 min). Our analysis revealed that, on average, participants in the MCI group consistently surpassed the specified threshold during the “Hot Meal Preparation” activity. Consequently, the framework systematically identified issues within this group as a result of the activity duration rule (Rule 1). In contrast, for the HC group, the average duration for the “Hot Meal Preparation” activity was below the threshold. This resulted in the framework detecting significantly fewer problems, mainly observed among a few outlier participants deviating from the average. Moreover, participants in the SCD group were observed to slightly exceed the threshold on average, leading to the framework identifying duration issues in several participants, although not as consistently as in the MCI group.

In addition, the average number of events required by each participant to complete the “Hot Meal Preparation” activity was also evaluated. Based on the protocol, the maximum normal count of events for this activity was set to 20. Notably, participants in the MCI group exhibited an average step count slightly lower than the threshold. This led the framework to identify specific instances of step-related issues for participants in this group as a result of the count of events rule (Rule 3). Moreover, participants in both the HC and SCD groups demonstrated nearly equal average step counts for this activity, which were even lower than those observed in the MCI group. In these groups, only a few outlier participants deviated significantly from this average count, prompting the framework to raise an issue for them.

This comparison provides insights into the performance variations across different cognitive status groups, highlighting the effectiveness of the semantic framework in detecting deviations from expected activity duration.

Based on the detailed analyses of activity duration and step-related performance for participants in the HC, SCD, and MCI groups, we can now proceed to an overall assessment of problems detected by our semantic framework during the “Hot Meal Preparation” activity. Table 4 shows the percentage of participants from the three groups (HC, SCD, MCI) for whom our framework detected problems regarding the activity “Hot Meal Preparation”. A significant difference in the “Too Long Duration” problem is observed among the three groups. The activity duration rule (Rule 1) generated a problem for 18.18% of the HC participants, while almost half of the SCD participants (45.45%) and 62.5% of the MCI were detected with the same problem. The results related to protocol divergences are particularly interesting, given that 81% of both HC and SCD participants exhibited deviations, and all MCI participants did as well. These especially high percentages reveal that, despite the specific protocol instructions, most participants executed the activity in a more intuitive and subjectively natural way due to its simplicity and flexibility. Regarding the count of events rule (Rule 3), the framework detected the “Extra Steps” problem in 12.5% of participants from both the HC and SCD groups, with an increase to 37.5% in the MCI group. It is worth noting that the framework did not detect any “Missing Steps” problems in any of the participants from the three groups. This result may indicate that none of the participants quit the activity without successful completion.

This case scenario shows the ability of our framework to handle and process data from smart home sensors, translate them into meaningful representations of events and ADLs, and finally, detect problems in the execution of these ADLs. In this way, our framework can be used as a helpful tool for monitoring the deterioration of ADLs performance for an individual by collecting more long-term observations for each participant of the study. These results can be used as an alert mechanism warning the clinician when there is deterioration of the participant’s performance in executing ADLs.

## 5. Conclusions—Future Work

In this paper, we present our semantic approach to detect problems in ADLs execution that are monitored through smart home sensors. The proposed framework combines different Semantic Web technologies (i.e., ontology, triplestore, SPARQL) to handle and transform smart home sensor data into meaningful representations, forming a knowledge graph. The main purpose of our framework is to detect problematic conditions in ADLs execution by applying predefined rules to the generated knowledge graph. The main architecture of this framework and its components were presented in detail. In order to validate our system, we have also provided a proof-of-concept case scenario that utilizes real-life data from participants who took part in the smart home study.

It is of high importance that our framework offers an easy-to-comprehend way for clinicians to describe the problematic incidents that are observed in a patient. Instead of observing a generic functional and cognitive decline, it can specify the actual problems relying on patients’ data. In addition, it is a modular framework that can easily scale up with more features, activities and problems while preserving the integrity of its core logic.

In future work, we plan to enrich the problematic situations that this framework can detect by defining more rules evaluating different and more complicated aspects of ADLs performance. Moreover, we aim to adjust it to support input data from other similar IoT sensors that are available in the market. Our intention is for our framework to be adopted by clinicians as an objective measure for detecting the progression of cognitive decline in an unobtrusive and natural way.

## 6. Limitations

Under the study’s limitations, the small sample size is noted. While the inclusion of forty participants can be considered satisfactory given the study’s exploratory nature, as not all participants completed all three tasks, this led to a further decrease in the dataset.

Moreover, an assumption has been made that the observed differences in performing various ADLs are a direct result of the participants’ cognitive state and can be linked to functional deterioration, not taking into account that a person may be performing an ADL faster or slower than another. As no baseline data were collected prior to the participants’ daily visit to the Smart Home, it is not possible to know how the participants usually perform various ADLs.

Additionally, the proposed approach would need to be further developed to address other scenarios. For example, the approach presented herein could be utilized in a longitudinal study to assess the potential functional deterioration of individual users. As the ADLs performed even by the same user would exhibit important variability, baseline data would need to be collected and assessed in order to identify personalized thresholds.

Furthermore, it is noted that the proposed framework explores the performance of individuals and has no current application in a scenario where multiple individuals coexist. However, further development and research could hold potential as there is still valuable information to be obtained and alternative outcomes and problems to be explored. For example, in [46], activities observed are classified on “a household level”, and differences can be attributed to homes where individuals with MCI cohabit.

Finally, it is recognized that manual activity recognition is an intricate process that may be prone to errors. Further exploration of automated approaches, utilizing ontology and reasoning, is essential to improve the accuracy and reliability of activity recognition within the framework.

## Figures and Tables

**Table 1:** Set of devices installed in the smart home.

#	Device	Quantity	Description	Data Type
1	Motion sensor	8	Presence/motion capture	Boolean (0,1)
2	Door/drawer/cabinet sensor	8	Door/drawer/cabinet opening—closing	Boolean (0,1)
3	Panic button	4	Emergency alarm triggering	Boolean (0,1)
4	Wall plugs	6	Consumption monitoring	Floating-point number

**Table 2:** Rule table for problem detection.

#	Variables	Rule	Problem
1	activity duration	activity duration > threshold	Activity takes too long
2	order of events	given order != correct order	Divergence from protocol
3	count of events	given count != correct count	Took extra steps
4	count of distinct events	given count < correct count	Missing steps

**Table 3:** Comparison of average duration and steps for ’Hot Meal Preparation’ activity among the three cognitive status groups.

	HC (N = 13)	SCD (N = 14)	MCI (N = 13)
Avg Duration (seconds)	1782.36	2179.81	2399
Avg Steps (counts)	15.72	15.63	18.37

**Table 4:** Problems detected for the activity “Hot Meal Preparation”.

	Too Long Duration (%)	Divergence (%)	Extra Steps (%)
HC (N = 13)	18.18	81	12.50
SCD (N = 14)	45.45	81	12.50
MCI (N = 13)	62.50	100	37.50

## Data Availability

The data presented in this study are available on request from the corresponding author.

## Abbreviations

The following abbreviations are used in this manuscript:

ADLs	Activities of Daily Living
BADLs	Basic Activities of Daily Living
IADLs	Instrumental Activities of Daily Living
AD	Alzheimer’s Disease
RDF	Resource Description Framework
OWL	Web Ontology Language
IoT	Internet of Things
HC	Healthy Controls
SCD	Subjective Cognitive Decline
MCI	Mild Cognitive Impairment
SSN	Semantic Sensor Network
SOSA	Sensor, Observation, Sample, and Actuator
